# Characterization of the mitochondrial genome of the plain flowerpecker, *Dicaeum concolor* (Dicaeidae) from Yunnan Province, China

**DOI:** 10.1080/23802359.2021.1914525

**Published:** 2021-06-29

**Authors:** Yong Gao, Huanhuan Chen, Si Yin, Lei Zhu

**Affiliations:** College of Biological Resource and Food Engineering, Qujing Normal University, Qujing, China

**Keywords:** Plain flowerpecker, Dicaeum concolor, mitochondrial genome, phylogenetic analysis

## Abstract

The plain flowerpecker, *Dicaeum concolor*, is a tiny bird classified in the flowerpecker family Dicaeidae, and is an important pollinator and disperser of mistletoes in forests. This species is distributed across South and Southeast Asia. For further phylogeographic studies of *D. concolor*, the complete mitochondrial genome was sequenced here. The genome (GenBank accession no. MW429430) is 16,796 bp in length and comprises 13 protein-coding genes, 22 transfer RNAs, and two ribosomal RNAs. The GC content of the genome sequence is 45.46%, with an overall base composition of 30.76% A, 30.92% C, 14.53% G, and 23.79% T. A maximum-likelihood analysis placed *D. concolor* as sister relationship to *D. eximium*. The mitochondrial genome of the plain flowerpecker will be useful for studies of molecular evolution in flowerpeckers.

The plain flowerpecker, *Dicaeum concolor*, is a tiny bird in the Dicaeidae family (Cheke and Mann 2008). Like other family members, it feeds predominantly on nectar and fruits (Luo et al. 2016). An important pollinator and disperser of mistletoes, plain flowerpeckers forage within the forest canopy and are found across South and Southeast Asia (Cheke and Mann 2008). This species is non-migratory and non-overlapping, forming morphologically distinct populations that occur across its range, some of which are recognized as distinct subspecies (Sheldon 1985). To facilitate the phylogeographic and systematic research of this species, the complete mitochondrial DNA sequence of *D. concolor* was deciphered.

One plain flowerpecker was captured from Mengla (E 101°32'28", N 21°27'34.2"), in Yunnan Province, China. A blood sample was taken from the brachial vein and then stored in a −80 °C freezer at the herbarium of College of Biological Resources and Food Engineering, Qujing Normal University (voucher no. QJNU-Zhu-20200801, Lizhou Tang, biologytang@163.com). Total genomic DNA was extracted using a commercial blood DNA isolation kit (DP318; TIANGEN, Beijing, China). One microgram of DNA was used to create a sequencing library with the VAHTS Universal DNA Library Prep Kit for MGI (Vazyme, Nanjing, China), following the manufacturer's recommendations. DNA sequencing was performed on a MGI-SEQ 2000 platform by Frasergen Bioinformatics Co., Ltd. (Wuhan, China). NOVOPlasty (Nicolas et al. 2017) and MITOS (Bernt et al. 2013) were then utilized for mitogenome assembly and annotation, respectively. Mitochondrial genome sequences for ten other species in the superfamily Passeroidea were downloaded from NCBI, for the phylogenetic analysis. The complete mitochondrial genome of *Nucifraga caryocatactes* was also downloaded and designated as the outgroup. DNA sequences were aligned using MUSCLE (Edgar 2004) and a maximum-likelihood (ML) tree was created with MEGA 7 (Kumar et al. 2016) using the general time reversible model (GTR) with 1000 bootstrap replicates.

The length of the sequenced mitochondrial genome of *D. concolor* (GenBank accession no. MW429430) was 16,796 bp. The genome sequence contained 13 protein-coding genes, 22 transfer RNAs, and two ribosomal RNAs. The GC content was 45.46% and the overall base composition: 30.76% A, 30.92% C, 14.53% G, and 23.79% T. In the ML tree, *D. concolor* was fully resolved in a clade with *D. eximium*. A close relationship between *Dicaeum* and *Leptocoma* was also suggested by the phylogenetic tree (Figure 1). The mitochondrial genome of *D. concolor* provides an important resource for molecular evolution and phylogeographic studies.

**Figure 1.**
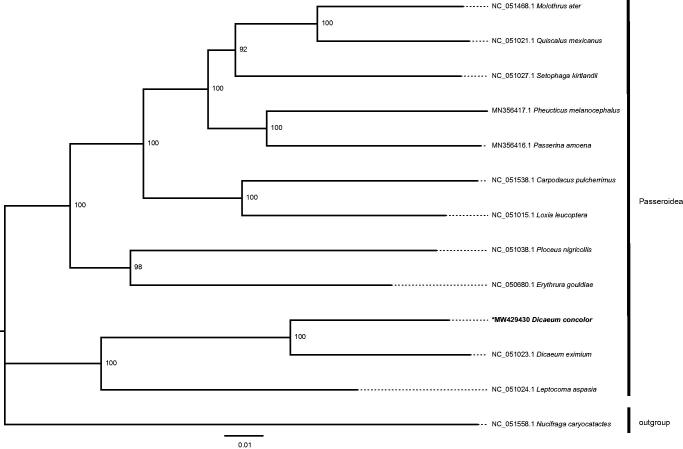
The maximum-likelihood (ML) tree constructed using mitochondrial genome sequences from *Dicaeum concolor* and twelve other Passeroidea species; *Nucifraga caryocatactes* was used as the outgroup. Bootstrap values based on 1000 replicates are shown for each node.

## Data Availability

The genome sequence data that support the findings of this study are openly available in GenBank of NCBI at (https://www.ncbi.nlm.nih.gov/) under the accession no. MW429430. The associated BioProject, SRA, and Bio-Sample numbers are PRJNA706879, SRR13862305, and SAMN18147082 respectively.
